# The Book World of Medicine and Science

**Published:** 1897-09-18

**Authors:** 


### THE BOOK WORLD OF MEDICINE AND SCIENCE.

EYE-STRAIN IN HEALTH AND DISEASE. With Special Reference to the Amelioration or Cure of Chronic Nervous Derangements Without the Aid of Drugs. By AMBROSE L. RANNEY, A.M., M.D., late Professor of Nervous Diseases in the Medical Department of the University of Vermont and of the Anatomy of the Nervous System in the New York Post-Graduate Medical School, &c. Illustrated with 38 woodcuts. (Philadelphia: The F. A. Davis Co. Pp. 321. Price $2.00 net.)

This is a forcibly-written book, and we commend it to those who are desirous of knowing the extreme views with regard to the possible deleterious effects of eye-strain which have recently been put forward. The author includes under the term "eye strain" efforts to overcome refractive errors, and efforts to adjust the improper balance of muscles which move the eyes. He considers that the following affections may be caused by it: Headache, neuralgia, chorea, nervous prostration, insanity, sleeplessness, chronic gastric and digestive disturbances, epilepsy, styes, enlargement of the meibomian glands, blepharitis, chronic conjunctivitis, corneal ulcers, episcleritis, pterygium, epiphora, ptosis, glaucoma, cataract, retinitis, and optic neuritis. He further considers that it may favour the development of a tubercular or gouty tendency. Hetrophoria, or want of balance of the muscles which move the eyes, is an exceedingly common condition, and before it can be proved that any of the above diseases are caused by it, we must ascertain if it is more often met with or is present in higher degrees in patients suffering from them than in the unaffected. Dr. Ranney has not attempted to do this; he contents himself with recording in considerable detail cases of the various diseases in which "eye-strain" was present, and which he successfully treated with glasses and limited tenotomies. As an example of the small defect in ocular balance which Dr. Ranney believes may give rise to such a disease as epilepsy we may quote briefly the following case: A woman, aged 30, was the subject of frequent epileptic seizures commencing after parturition. On examination of her eyes no error of refraction could be discovered, and there was no hetrophoria, only slight defect in the power of abduction. Esophorial prisms were ordered for 24 hours; "latent esophoria" then became manifest. A limited tenotomy was performed. Since the operation the epileptic seizures have ceased. The true significance of want of balance in the ocular muscles in connection with functional nervous disorders and ocular affections is a subject requiring further investigation. But the inquiry must be carried out in a more impartial spirit than that in which Dr. Ranney's book is written.

